# Thermoresponsive motor behavior is mediated by ring neuron circuits in the central complex of *Drosophila*

**DOI:** 10.1038/s41598-020-80103-9

**Published:** 2021-01-08

**Authors:** Edgar Buhl, Benjamin Kottler, James J. L. Hodge, Frank Hirth

**Affiliations:** 1 School of Physiology, Pharmacology and Neuroscience, University of Bristol, University Walk, Bristol, UK; 2 Department of Basic and Clinical Neuroscience, Institute of Psychiatry, Psychology and Neuroscience, King’s College London, London, UK

**Keywords:** Sensorimotor processing, Neural circuits, Patch clamp, Drosophila, Fluorescence imaging, Ca2+ imaging, Confocal microscopy, Neurophysiology

## Abstract

Insects are ectothermal animals that are constrained in their survival and reproduction by external temperature fluctuations which require either active avoidance of or movement towards a given heat source. In *Drosophila*, different thermoreceptors and neurons have been identified that mediate temperature sensation to maintain the animal’s thermal preference. However, less is known how thermosensory information is integrated to gate thermoresponsive motor behavior. Here we use transsynaptic tracing together with calcium imaging, electrophysiology and thermogenetic manipulations in freely moving *Drosophila* exposed to elevated temperature and identify different functions of ellipsoid body ring neurons, R1-R4, in thermoresponsive motor behavior. Our results show that warming of the external surroundings elicits calcium influx specifically in R2-R4 but not in R1, which evokes threshold-dependent neural activity in the outer layer ring neurons. In contrast to R2, R3 and R4d neurons, thermogenetic inactivation of R4m and R1 neurons expressing the temperature-sensitive mutant allele of dynamin, *shibire*^*TS*^, results in impaired thermoresponsive motor behavior at elevated 31 °C. *trans*-Tango mediated transsynaptic tracing together with physiological and behavioral analyses indicate that integrated sensory information of warming is registered by neural activity of R4m as input layer of the ellipsoid body ring neuropil and relayed on to R1 output neurons that gate an adaptive motor response*.* Together these findings imply that segregated activities of central complex ring neurons mediate sensory-motor transformation of external temperature changes and gate thermoresponsive motor behavior in *Drosophila*.

## Introduction

Animals constantly monitor environmental conditions and integrate this information with their internal state in order to adapt their behavior and maximise fitness. This is particularly evident for ectothermal animals that are constrained by temperature conditions in which they can survive and function. For example, insects exposed to direct sunlight can heat up by 10 °C within seconds^1^, which can trigger swift movements to relocate to an ambient surrounding fitting their thermal preference. Pioneering studies in *Drosophila* showed that fruit flies prefer surround temperature around 24 °C, which is dependent on proper thermosensation^2^. The perception of temperature is initiated by the activation of thermo-sensors belonging to a large, highly conserved protein family of transient receptor potential (TRP) ion channel^3^. In addition to TRPs, other channel receptors like GR28b that differ in their relative activation threshold, convey thermosensory information onto thermoreceptor neurons (reviewed in Refs.^4–6^). In *Drosophila*, several thermoreceptor neurons have been identified, including Anterior Cells (AC)^7^ and Hot Cells (HC)^8^, that integrate surround temperature^6,9,10^ in the proximal antennal protocerebrum and the posterior antennal lobe, which forms a sensory map for hot and cold that together aid thermotaxis and nociception^8,11–13^.

Despite growing knowledge into thermosensation, insights into the neural circuits required for sensory-motor transformation are only starting to emerge. In *Drosophila*, the mushroom bodies have been implicated in thermal preference behavior^14,15^. Warm-sensitive AC neurons project to the antennal lobe, the subesophageal ganglion, and the superior lateral protocerebrum^7^ and also to PDF-expressing neurons involved in the circadian clock^16^; however the roles of these brain regions in regulating thermal preference behavior are largely unknown^17^. Moreover, it has remained elusive how thermosensory information is integrated to gate thermoresponsive motor behavior.

**Figure 1:**
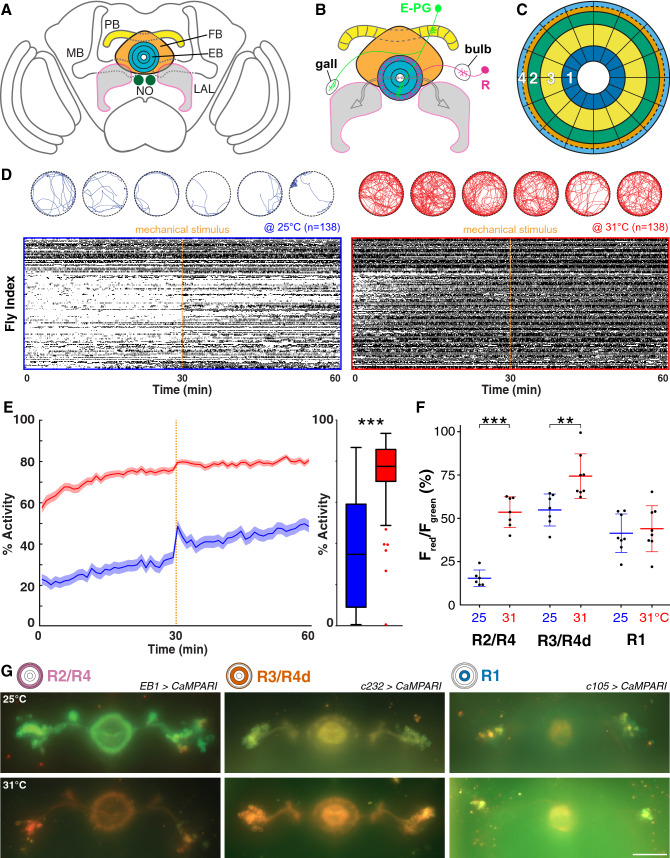
Increasing temperature causes thermoresponsive motor behavior and Ca^2+^ influx in R1-R4 ring neurons in *Drosophila*. (**A**) Cartoon of adult *Drosophila* brain showing central complex (CX) ground pattern (PB, protocerebral bridge; FB, fan-shaped body; EB, ellipsoid body; NO, noduli; LAL, lateral accessory lobes—MB, mushroom bodies are shown for orientation). (**B**) Two major types of projection neurons characterize the circuit architecture of the CX, columnar neurons (example shown, Ellipsoid body-Protocerebral bridge-Gall neuron, E-PG in green) and tangential neurons (example shown, EB ring neuron, R in red); CX neuropils depicted as in A, in addition to gall and bulb. (**C**) The EB forms a toroidal or ring-like neuropil that resembles a 'closed arch'. Projections from tangential R neurons divide it into at least 4 different layers (white numbers 1–4), R1-R4. Sensory associations from PB and FB converge onto EB modules that can be subdivided according to PB-FB input into segments of sensory space, spanning the left and right hemispheres. (**D**) Raster plots showing sequences of activity (black bar) and inactivity (white spaces in-between) of 138 wild-type flies recorded either at 25 °C (left panel surrounded by blue frame) or 31 °C (right panel, red frame); vertical dashed orange line indicates mechanical stimulation. Above, trajectories shown for 6 exemplary flies recorded for the first 10 min either at 25 °C (in blue) or 31 °C (in red). (**E**) Flies show a significant increase of activity in response to elevated temperature across the whole 60 min recording of the experiment, including the response to mechanical stimulation given after 30 min (orange dashed line). Solid line, mean; shaded area, SEM; box and whiskers, median, IQR, Tukey. (**F**,**G**) Fluorescence after photoconversion of R2/R4 (*EB1-Gal4*), R3/R4d (*c232-Gal4*) and R1 (*c105-Gal4*) EB ring neuropil and neurons expressing CaMPARI at 25 °C (**G**, top row) and 31 °C (bottom row) respectively and (**F**) quantification of the red/green ratio shows a Ca^2+^ increase for R2/R4 and R3/R4d only. Scale bar, 50 µm, mean ± SD, n ≥ 6. **p < 0.01, ***p > 0.001, Mann–Whitney test for (**E**), one-way ANOVA with Sidak’s post hoc test for (**F**). See also Supplementary Figs. S1 and S2.

In flies and other insects, several lines of evidence suggest that the central complex (CX) integrates various sensory cues and orchestrates motor output for adaptive behavior^18,19^. The CX is a central brain structure composed of midline neuropils comprising the protocerebral bridge (PB), the fan-shaped body (FB), the ellipsoid body (EB), and the noduli, together which are connected to the lateral accessory lobes (LAL) that are part of the lateral complex^20–23^ (Fig. 1A). Previous studies implicated the CX in the higher control of behavior, including locomotion, orientation and courtship behavior, visual memory and place learning as well as attention, arousal and decision-making^24–37^. Although triggered by different sensory modalities, the gating of these behaviors involves two major types of projection neurons that characterize the circuit architecture of the CX (Fig. 1B).

Columnar neurons interconnect the different substructures of the CX, from PB to EB^20,22,23,38,39^, and compartmentalize them into modules, each of which corresponds to a segment of sensory space^40^. Tangential neurons form synaptic layers of the FB and EB that intersect columnar projections thereby generating a grid-like pattern of sensory space^40^. This is particularly evident for CX ring neurons that can be distinguished into different subtypes based on their layer-specific projections that form the toroidal EB neuropil^20,23,39,41–43^ (Fig. 1C). Ring neurons are classified into at least four subtypes, R1-R4 (sometimes also R5) each resolved by subtype-specific *Gal4* lines^42,43^. Among other functions, ring neurons have been shown to relay visual cues via the bulbs to the EB ring neuropil^35^, to regulate visual place learning and visual orientation memory^31–33,44^, and to mediate sleep, arousal and turning behavior^29,45,46^. These data suggest that EB ring neuron circuitry integrates multiple modalities of external and self-generated sensory cues to gate adaptive motor behavior. We therefore asked whether EB ring neuron circuits might also be involved in sensory-motor transformation of integrated temperature cues to gate thermoresponsive motor behavior. Here we show that differential activities of EB ring neuron circuits mediate sensory-motor transformation as part of the regulatory network underlying thermoresponsive behavior in *Drosophila*.

## Results

### Increasing temperature causes thermoresponsive motor behavior in *Drosophila*

To investigate the role of EB ring neurons in thermoresponsive motor behavior, we made use of earlier observations which showed that an increase of surround temperature from the preferred 24–31 °C evokes goal-directed locomotion^2^. To quantify behavioral activity, we measured locomotion using an open-field assay, employing video-assisted motion tracking to record freely moving *Drosophila*^45^. Flies were recorded for 60 min in 35 mm diameter arenas either at 25 °C or 31 °C, with a short pulse of mechanical stimulation applied after 30 min (Fig. 1D and methods) to assess their arousal^47^. Based on these recordings, we generated raster plots and trajectories for each individual fly and calculated their overall activity. We extracted parameters detailing motor behavior including the average walking speed, the number of initiated activity bouts (action initiation), the length of walking bouts and the response to sensory stimulation (Supplementary Fig. S1). In addition, we measured the duration of pauses (interbout interval, IBI) and determined a Weibull distribution and its shape factor κ that calculates the distribution of IBIs over time as a measure for random walks or burstiness^45^ (Supplementary Fig. S2).

In response to 31 °C surround temperature, control flies more than doubled their motor activity throughout the recording period (from 34 to 77%, Fig. 1E); they walked faster (from 6.2 to 10.4 mm/s) and initiated more (from 0.3 to 1.1 initiations/s) and longer (from 1.6 to 3.2 s) walking bouts (Supplementary Fig. S1). Interbout-intervals became shorter (IBI, from 3.2 to 0.9 s), with the cumulative distribution of IBIs fitted on a Weibull function revealing a higher shape factor κ (from 0.27 to 0.45), demonstrating that IBIs were more randomly distributed (Supplementary Fig. S2). In response to a mechanical stimulus, flies increased their walking speed in both 25 °C (2.4 mm/s) and 31 °C (1.7 mm/s) recording conditions, compared to before the stimulus. However, this response was less pronounced, but not significantly so at 31 °C, likely because flies were already walking faster before the stimulus was applied (Supplementary Fig. S1). Consistent with earlier reports^2^, these data demonstrate that an increase in environmental temperature to 31 °C evokes thermoresponsive motor behavior in freely moving *Drosophila*.

### Increasing temperature causes ring neuron subtype-specific calcium influx

Next, we investigated whether EB ring neurons are involved in processing integrated temperature information. Since none of the known thermal receptors are expressed in the EB^5,6^, we first asked whether integrated temperature information is received and processed by EB ring neurons. For this, we used *EB1-*, *c232-* and *c105*-*Gal4* drivers to express *UAS*-transgenes for functional imaging and thermogenetic manipulations targeted to R2/R4, R3/R4d and R1 neurons, respectively. We tested for a temperature-related functional response and utilised the fluorescent ratiometric calcium (Ca^2+^) sensor CaMPARI^48^. For this, animals were kept at either 25 °C or 31 °C for 30 min prior to photoconversion (see methods). The resulting red-to-green ratio showed different baselines at 25 °C for the different EB ring neuron subtypes, probably caused by different amounts of free intracellular Ca^2+^ indicating different neuronal activity (Fig. 1F,G). The lowest ratio was recorded for R2/R4 neuron-specific *EB1* > *CaMPARI* with 16%, while the higher values for R1 neuron-specific *c105* > *CaMPARI* (42%) and R3/R4d neuron-specific *c232* > *CaMPARI* (55%) suggest higher amounts of free intracellular Ca^2+^ in these structures caused by more active neurons. Photoconversion after 30 min at 31 °C showed a significant 38% increase in R2/R4 neurons when compared to 25 °C, followed by a 20% increase in R3/R4d, whereas R1 neurons only showed a 2.5% increase suggesting they did not respond to elevated temperature (Fig. 1F). These results demonstrate that an increase in surround temperature to 31 °C evokes a strong Ca^2+^ response in EB1-specific R2/R4 neurons and a somewhat weaker response in R3/R4d neurons, but no response in R1 neurons. These data suggest that EB ring neurons process temperature information in a graded, layer-specific outside-in response.

### R2/R4 and R1 ring neurons mediate thermoresponsive motor behavior

Our results complement previous findings, which show that EB ring neurons integrate multiple modalities of external sensory cues to gate adaptive motor behavior^18,19,49–51^. Accordingly, the observed temperature-related activity increase suggests that ring neurons, especially R2/R4, might be involved in sensory-motor transformation to gate thermoresponsive motor behavior. To test this hypothesis, we thermogenetically manipulated ring neurons by targeted expression of the temperature-sensitive mutant allele of dynamin, *shibire*^*TS*^ ^52^. *Gal4*-mediated expression of *UAS-shibire*^*TS*^ has been shown to block vesicle endocytosis above 25 °C, and to modify behavior at 31 °C^53^. Thus, we investigated whether flies expressing *UAS-shibire*^*TS*^ in specific ring neuron subtypes would be affected in their thermoresponsive motor behavior.

**Figure 2:**
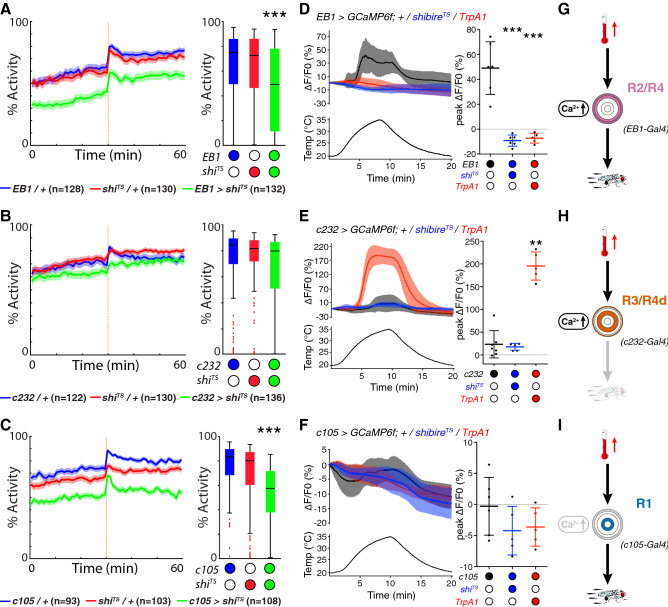
Segregated ring neuron activities mediate thermoresponsive motor behavior in *Drosophila*. (**A**) R2/R4—*EB1* > *shibire*^*TS*^ flies show reduced thermoresponsive motor behavior. Average fly locomotor activity over 60 min recordings at 31 °C; orange dashed line indicates mechanical stimulation at 30 min; *shibire*^*TS*^ (green) compared to *Gal4* (blue) and *UAS* controls (red). Solid line, mean; shaded area, SEM; box and whiskers, median, IQR, Tukey. (**B**) Recorded at 31 °C, activity levels of R3/R4d—*c232* > *shibire*^*TS*^ flies (green) are comparable to *Gal4* (blue) and *UAS* (red) controls. (**C**) R1—*c105* > *shibire*^*TS*^ flies (green) show reduced thermoresponsive motor behavior, compared to *Gal4* (blue) and *UAS* (red) controls. (**D**) Average (left) and peak (right) fluorescence GCaMP imaging of *shibire*^*TS*^ (blue) and *TrpA1* (red) R2/R4—*EB1* > *GCaMP6f* brains to temperature ramps from 20 °C to 35 °C show no response compared to controls (black). Mean ± SD, n ≥ 5. (**E**) R3/R4d—*c232* > *GCaMP6f* Ca^2+^ signals co-expressing either *shibire*^*TS*^ (blue) or *TrpA1* (red) during temperature ramps from 20 to 35 °C reveal a massive increase with *TrpA1* compared to only a small activation for controls and with *shibire*^*TS*^. Mean ± SD, n ≥ 5. (**F**) GCaMP imaging shows no temperature response in any condition for R1—*c105* > *GCaMP6f*. Mean ± SD, n ≥ 5. (**G**) R2/R4 neurons integrate temperature increase and mediate thermoresponsive motor behavior. (**H**) R3/R4d neurons integrate temperature increase but do not mediate thermoresponsive motor behavior. (**I**) R1 neurons do not integrate temperature increase but mediate thermoresponsive motor behavior. **p < 0.01, ***p < 0.001, Kruskal–Wallis with Dunn’s post hoc test for (**A**,**C**,**E**), one-way ANOVA with Dunnett’s post hoc test for (**D**). See also Supplementary Figs. S1, S2 and S3.

In comparison to genetic controls (*Gal4/*+ and *UAS/*+), recordings at 31 °C revealed a reduction in activity of R2/R4—*EB1* > *shibire*^*TS*^ flies to 49%, close to levels seen at 25 °C (Fig. 2A and see Fig. 1E). These flies also walked slower (8.0 mm/s), initiated fewer (0.5 starts/s) and shorter (1.8 s) walking bouts at longer intervals (2.1 s) with a reduced shape factor κ (0.22) while the response to stimulation was only slightly reduced (1.9 mm/s), as shown in Supplementary Figs. S1 and S2. We then tested R3/R4d ring neurons for their involvement in thermoresponsive motor behavior. However, *c232-Gal4* mediated expression of *UAS-shibire*^*TS*^ did not affect the overall activity levels of R3/R4d—*c232* > *shibire*^*TS*^ flies recorded at 31 °C (Fig. 2B), except for a slightly decreased walking speed (8.7 mm/s) and a decreased shape factor κ (0.26) (Supplementary Figs. S1 and S2). In contrast, analysis of the temperature-induced locomotor behavior of R1—*c105* > *shibire*^*TS*^ flies recorded at 31 °C (Fig. 2C) revealed a significant reduction in activity to 57%, a decreased walking speed (6.8 mm/s), fewer initiations (1.1 s^−1^), shorter (1.3 s) and more spaced (0.9 s) activity bouts with a smaller κ (0.27) but unaffected startle response (2.4 mm/s) (Supplementary Figs. S1 and S2). Together these data demonstrate that R2/R4 as well as R1, but not R3/R4d ring neurons mediate thermoresponsive motor behavior.

### R2/R4 ring neurons integrate increasing temperature cues

Ring neuron-specific Ca^2+^ influx has been used as a proxy to measure their neuronal activity in response to an external sensory cue^35^. Thus, we carried out ex vivo activity-related GCaMP Ca^2+^-imaging^54^ of ring neurons in response to a graded temperature ramp from 20 to 35 °C and back to 20 °C (Fig. 2D–F and Supplementary Fig. S3). Similar to the observations with the fluorescent ratiometric Ca^2+^ sensor CaMPARI, GCaMP imaging revealed a response for R2/R4—*EB1* > *GCaMP6f* and for R3/R4d—*c232* > *GCaMP6f* only. R3/R4d neurons responded like R2/R4 neurons to increasing temperature but reached peak fluorescence at the maximum imposed 35 °C when signals of R2/R4 neurons were already decreasing (Supplementary Fig. S3). With 23%, the average peak change of R3/R4d was merely half of that for R2/R4 (49%). In contrast, R1—*c105* > *GCaMP6f* did not respond to temperature changes (0%, Supplementary Fig. S3), consistent with the CaMPARI data (see Fig. 1F). Thus, R2/R4 neurons quickly and strongly respond to an increase in surround temperature, with R3/R4d neurons responding less strongly and later, while R1 neurons do not respond.

Together these findings suggest that segregated ring neuron activity mediates sensory-motor transformation of integrated temperature cues to gate thermoresponsive motor behavior. To gain further insights into the role of ring neuron subtypes in this process, we carried out GCaMP imaging in response to increasing temperature, while simultaneously expressing either *UAS-shibire*^*TS*^ to block synaptic output or *UAS-TrpA1* to activate neurons^7,55^.

Compared to *EB1* > *GCaMP6f*; + controls, recording of R2/R4—*EB1* > *GCaMP6f; shibire*^*TS*^ and of *EB1* > *GCaMP6f*;* TrpA1* somewhat surprisingly revealed that neither activation with *TrpA1* (− 7% peak ΔF/F0 fluorescence) nor blocking synaptic output with *shibire*^*TS*^ (− 7% peak ΔF/F0 fluorescence) led to a Ca^2+^ response when heating the preparations to 35 °C (Fig. 2D). We then measured the GCaMP response of c232-specific R3/R4d neurons which revealed that compared to *c232* > *GCaMP6f*; + controls, recording of *c232* > *GCaMP6f*;* shibire*^*TS*^ detected no significant signal alteration (18% peak ΔF/F0 fluorescence). However, *UAS-TrpA1* expression resulted in a striking increase of 196% peak ΔF/F0 fluorescence caused by the activation of the cation channel at higher temperature (Fig. 2E). In contrast to R2/R4 and R3/R4d neurons, c105-specific R1 neurons did not respond to increasing temperature with a change in their Ca^2+^ response, neither for *c105* > *GCaMP6f; shibire*^*TS*^ (− 4% peak ΔF/F0 fluorescence) nor for *c105* > *GCaMP6f; TrpA1* (− 4% peak ΔF/F0 fluorescence) (Fig. 2F). Consistent with CaMPARI imaging (Fig. 1F,G), these data suggest that R2/R4 (Fig. 2G) as well as R3/R4d (Fig. 2H), but not R1 neurons (Fig. 2I), integrate temperature increase to 35 °C. In contrast to c232-specific R3/R4d neurons though, R2/R4 neurons also mediate thermoresponsive motor behavior, suggesting a key role of EB1-targeted ring neurons in sensory-motor transformation.

### Increasing temperature evokes neural activity in R2/R4 ring neurons

**Figure 3:**
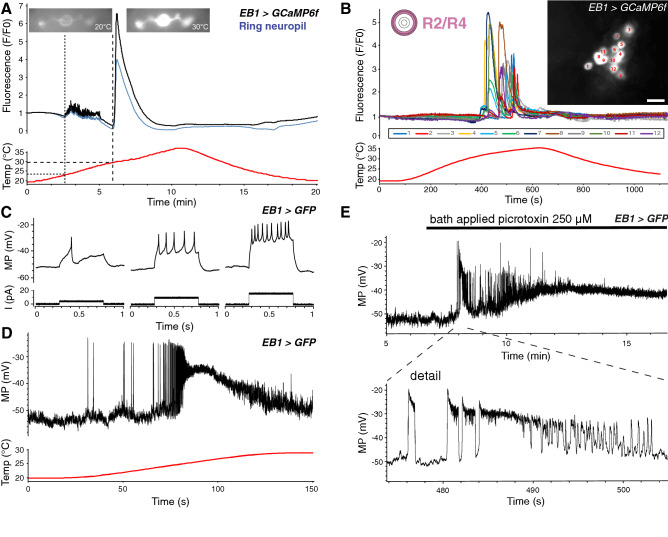
Increasing temperature evokes neural activity in inhibitory GABAergic R2/R4 ring neurons. (**A**) GCaMP imaging of an exemplary EB1-targeted R2/R4 ring neuron during a temperature ramp from 18 °C to 35°. Note that sparse activity starting at 23 °C (tight dashed line) resolves in a Ca^2+^ spike around 30 °C (wide dashed line) for both cell bodies (black line) as well as ring neuropil (blue line). (**B**) GCaMP imaging of individual cell bodies (indicated by colours and numbers in insert) of R2/R4 ring neurons during a temperature ramp from 20 to 35 °C show a brief Ca^2+^ spike for all cells at high temperature. Scale bar, 10 µm. (**C**) Whole-cell recordings of *EB1* > *mCD8::GFP* R2/R4 neurons. Injection of increasing amounts of depolarising current (I) evokes repetitive firing, with spike frequency increase proportional to stimulus strength. (**D**) Temperature increase from 20 °C to 30 °C elicits an increase in activity of an EB1-targeted R2/R4 ring neuron up to depolarising block before hyperpolarisation towards rest. (**E**) Spiking and bursting activity of an EB1-targeted R2/R4 ring neuron after application of 250 µM picrotoxin. MP, membrane potential; I, current. See also Supplementary Fig. S3 and Supplementary Video SV1.

In order to further examine the kinetics of the temperature response, we studied the response of R2/R4—*EB1* > *GCaMP6f* brains in more detail (Fig. 3A). The GCaMP signal of an exemplary brain revealed a saccade of small peaks starting around 23 °C, indicative of moderate activation of individual neurons, which by > 30 °C coalesced in a fluorescence spike in both R2/R4 cell somas and ring neuropil. Spiking occurred across the whole population with individual *EB1* > *GCaMP6f* labelled neurons responding at slightly different temperatures and with varying intensity above 27 °C (Fig. 3B and Supplementary Video SV1). GCaMP fluorescence intensity increased 4- to 7-fold above baseline for ring neuropil and cell bodies, respectively, and rapidly declined over 2 min, followed by inactivity even at noxious 35 °C (Fig. 3A).

To corroborate these findings and to gain further insights into the physiological response of R2/R4 neurons, we carried out ex vivo electrophysiological whole-cell patch recordings of *EB1* > *mCD8::GFP*-labelled R2/R4 neurons. This revealed resting membrane potentials of − 52.9 ± 4.0 mV (mean ± standard deviation, SD) and input resistances of 1185 ± 407 MΩ (n = 20). Injection of increasing amounts of depolarising current (up to + 40 pA) evoked repetitive firing with successively more spikes triggered by higher currents, up to a maximum frequency of 80 Hz (Fig. 3C), suggesting that spike frequency increased proportional to stimulus strength. We then measured the response of EB1-specific R2/R4 neurons to a temperature increase from 20 to 30 °C. These recordings demonstrated a physiological response that mimicked the activity-related Ca^2+^-imaging for three out of four tested neurons (Fig. 3D, compare with Fig. 3A). Detailed observation of an exemplary EB1-targeted ring neuron showed it was silent at rest but responded to increasing temperature, first by a few individual spikes corresponding to the small Ca^2+^ peaks seen at lower temperature; second by more frequent spiking while the neuron depolarised, likely coinciding with a massive increase in intracellular Ca^2+^ as seen by the corresponding GCaMP spike; third by a period of depolarised block where no individual spikes were generated; and finally by a return to resting potential without further spiking (Fig. 3D). Thus, both whole-cell recordings and Ca^2+^-imaging demonstrate a robust response of R2/R4 neurons to increasing temperature.

### R2/R4 ring neurons are inhibitory GABAergic

Despite the fact that both Ca^2+^-imaging and electrophysiological recordings of R2/R4 neurons evoked neural activity in a time and temperature-related manner*,* neither *EB1* > *GCaMP6f; shibire*^*TS*^ nor *EB1* > *GCaMP6f; TrpA1 *ex vivo imaging showed Ca^2+^ responses upon temperature increase. These observations were remarkable in two ways. First, a GCaMP response could be expected in *EB1* > *GCaMP6f; TrpA1* flies because thermogenetic activation of TrpA1 cation channels can lead to increased intracellular Ca^2+^ ^7,55^; however, higher Ca^2+^ concentrations have also been shown to rapidly inactivate TrpA1^56^. Second, activation of *shibire*^*TS*^ impairs post-synaptic neurons^52^, however *EB1* > *shibire*^*TS*^ not only resulted in impaired behavioral output but also caused an abolished temperature-related GCaMP response in *EB1* > *GCaMP6f; shibire*^*TS*^ neurons themselves (Fig. 2D). These data suggest that R2/R4—*EB1-Gal4* targets a group of functionally distinct neurons that are connected by reciprocal inhibition^57^, thereby affecting each other’s activity^58,59^.

To investigate both possibilities, we first performed electrophysiological recordings to investigate whether R2/R4 neurons might be inhibitory GABAergic, as already suggested be earlier studies^20,60,61^. Following bath-application of picrotoxin (250 µM), which is known to block GABA-mediated inhibition^62^, all of the six recorded neurons of *EB1* > *mCD8::GFP*-labelled brains exhibited individual spikes followed by bursting activity that lasted between 10 and 40 s (Fig. 3E, n = 6). Bursts (0.5–10 Hz frequency) comprised up to 60 individual spikes, with an instantaneous spike frequency ranging from 30 to 100 Hz, followed by strong depolarisation which suppressed further activity. The pattern and time course of this picrotoxin response resembled the activity seen with Ca^2+^-imaging and electrophysiological recordings in response to increasing temperature (Fig. 3A,B,D), as well as for the response to increasing amounts of depolarising current (Fig. 3C).

**Figure 4:**
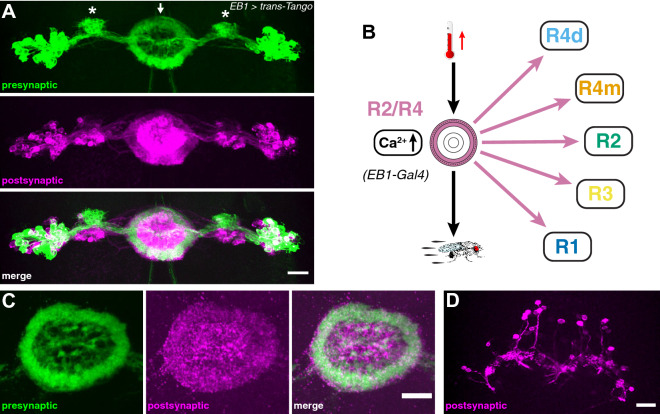
R2/R4 ring neurons connect to all EB rings. (**A**) *trans*-Tango based anterograde transsynaptic tracing of EB1*-*targeted R2/R4 ring neurons indicates connections onto R1–R4 ring neurons. Detail of the central brain showing presynaptic signal (top, green), the postsynaptic targets (middle, magenta) and merged image (bottom) of EB cell bodies and ring neuropil (arrowhead), as well as the bulb (asterisks). (**B**) R2/R4 neurons connect to all R1–R4 EB rings; they integrate temperature increase and mediate thermoresponsive motor behavior. (**C**,**D**) Details of the *trans-*Tango signal of EB1-targeted R2/R4 neurons showing (**C**) detail of the EB ring neuropil region with presynaptic (left, green), postsynaptic (middle, magenta) and merged signal (right) revealing connections to R1–R4 layers as well as (**D**) to columnar neurons and the protocerebral bridge. Scale bars, 20 µm.

We then examined the connectivity pattern of *EB1-Gal4* targeted R2/R4 neurons by utilizing the *trans*-Tango technique based on anterograde transsynaptic tracing^63^. *EB1* > *trans*-*Tango* flies identified R2/R4 ring neurons and layer-specific presynaptic projections to the EB ring neuropil (Fig. 4A, top in green and 4C). Postsynaptic *trans*-Tango labelling revealed connections to all ring neuron subtypes and neuropil layers (Fig. 4A, middle in magenta and 4B) which in many cases showed overlapping GFP and RFP immunolabelling (Fig. 4A, bottom). Of note, postsynaptic RFP-only patterns were detected for parts of the bulb (Fig. 4A, asterisks) as well as for columnar neurons and the protocerebral bridge (Fig. 4D), suggesting that R2/R4 activity reverberates with EB ring and columnar wedge neurons. These results suggest that R2/R4 neurons are inhibitory GABAergic interneurons that are connected to themselves and other R1–R4 neuron subtypes. Together with whole-cell recordings and Ca^2+^-imaging, these findings indicate that R2/R4 neurons assess sensory information, such as temperature increase, by threshold-dependent neuronal activity.

### R4m ring neurons mediate temperature integration and thermoresponsive motor behavior

**Figure 5:**
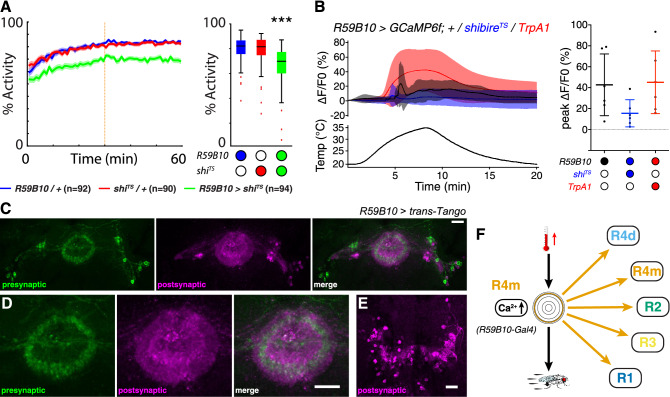
R4m ring neurons mediate temperature integration and thermoresponsive motor behavior. (**A**) Locomotor activity of R4m—*R59B10* > *Gal4* flies recorded at 31 °C (motor stimulation indicated by orange dashed line); R4m-specific output inactivation by *shibire*^*TS*^ (green) reduced temperature-induced locomotion compared to *Gal4* (blue) and *UAS* controls (red). Solid line, mean; shaded area, SEM; box and whiskers, median, IQR, Tukey. (**B**) Average (left) and peak (right) GCaMP responses of R4m—*R59B10* > *GCaMP6f* brains to temperature ramps from 20 to 35 °C for control (black), co-expressing *shibire*^*TS*^ (blue) or co-expressing *TrpA1* (red) reveals a similar peak Ca^2+^ influx for control and *TrpA1* compared to only a small influx for *shibire*^*TS*^. Mean ± SD, n = 6. (**C**) *trans*-Tango based anterograde transsynaptic tracing of R59B10*-*targeted R4m ring neurons with presynaptic signal (left, green), postsynaptic targets (middle, magenta) and merged image (right) of EB cell bodies and ring neuropil, as well as the bulb. (**D**,**E**) Details of *trans-*Tango labelling of R4m neurons indicate connections to (**C**) R1–R4 layers and (**D**) to columnar wedge neurons and the protocerebral bridge. (**F**) R4m neurons connect to all R1-R4 EB rings; they integrate temperature increase and mediate thermoresponsive motor behavior. ***p < 0.001, Kruskal–Wallis test with Dunn-Sidak correction for (**A**). Scale bars, 20 µm. See also Supplementary Figs. S1, S2 and S3.

**Figure 6:**
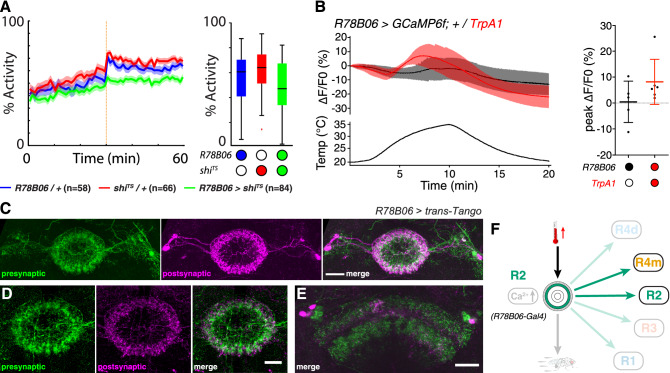
Connectivity and function of R2 ring neurons in thermoresponsive motor behavior. (**A**) Recorded at 31 °C, locomotor activity levels of R2—*R78B06* > *shibire*^*TS*^ flies (green) are comparable to *Gal4* (blue) and *UAS* (red) controls (motor stimulation indicated by orange dashed line). Solid line, mean; shaded area, SEM; box and whiskers, median, IQR, Tukey. (**B**) Average (left) and peak (right) GCaMP imaging shows no temperature response in either controls or co-expressed *TrpA1* for R2—*R78B06* > *GCaMP6f*. Mean ± SD, n ≥ 5. (**C**,**D**) *trans*-Tango transsynaptic tracing of R78B06*-*targeted R2 ring neurons reveals a reciprocal connection onto itself and to R4m. (**C**) Presynaptic signal (left, green), postsynaptic targets (middle, magenta) and merged image (right) of EB cell bodies and (**D**) detail of the ring neuropil. (**E**) *trans*-Tango imaging of *R78B06-Gal4* labels specific layers of the fan-shaped body (FB) both pre- and postsynaptically (merged image shown). (**F**) R2 neurons connect to R2 and R4m EB rings; they neither integrate temperature increase nor mediate thermoresponsive motor behavior. Scale bars, 20 µm. See also Supplementary Figs. S1, S2 and S3.

Reciprocal inhibition between GABAergic R2/R4 neurons could explain the abolished GCaMP response in *EB1* > *GCaMP6f; shibire*^*TS*^ and *EB1* > *GCaMP6f; TrpA1* neurons at increased temperature, by way of inhibiting their own output either by disinhibition (with *shibire*^*TS*^) or enhanced inhibition (with *TrpA1*). In addition, *EB1-Gal4* may target a group of functionally distinct neurons, thereby affecting each other’s activity^58,59^. To test this second possibility and to further disambiguate between R2 and R4 neurons, we made use of the Janelia *Gal4* collection^64^ and utilized two different *Gal4* lines, R59B10 specific to R4m neurons^65^ and R78B06 specific to R2 neurons^66^. To determine their role in sensory-motor transformation of increasing temperature, we performed behavioral experiments and GCaMP imaging together with *trans*-Tango labelling to investigate their connectivity pattern (Figs. 5, 6).

In comparison to genetic controls (*Gal4/*+ and *UAS/*+), recordings at 31 °C revealed a reduction in locomotor activity of R4m—*R59B10* > *shibire*^*TS*^ flies to 59% (Fig. 5A). These flies also walked slower (9.0 mm/s), showed fewer (1.3 initiations/s) and shorter walking bouts (1.7 s) at longer intervals (0.8 s) resulting in a smaller κ (0.37), however their response to a mechanical stimulus (− 1.2 mm/s) was largely unaltered (Supplementary Figs. S1 and S2). Ca^2+^-imaging revealed a 43% increased peak fluorescence temperature response for R4m similar in magnitude to EB1-targeted R2/R4 neurons, however, individual brain responses were short and peaking at different temperatures causing a wider spread of GCaMP peak ΔF/F0 fluorescence (Fig. 5B, compare to Fig. 2D and Supplementary Fig. S3). This peak fluorescence response was further reduced to 15% when co-expressing *UAS-shibire*^*TS*^. In contrast, *UAS-TrpA1* expression resulted in an increase to 45% ΔF/F0 fluorescence in *R59B10* > *GCaMP6f; TrpA1* preparations over a wide range of temperature (Fig. 5B). *R59B10* > *trans*-*Tango* identified R4m ring neurons and their layer-specific presynaptic projections to the EB ring neuropil (Fig. 5C, left), with postsynaptic *trans*-Tango labelling detectable in all ring neuron subtypes and neuropil layers (Fig. 5C, middle and right, and D, F), as well as in columnar wedge neurons and the protocerebral bridge (Fig. 5E).

In contrast to R59B10-specific R4m neurons, we did not detect any significant changes in Ca^2+^-imaging or thermoresponsive motor behavior when testing R2 neurons using *R78B06-Gal4*. Overall locomotor activity of *R78B06* > *shibire*^*TS*^ flies (Fig. 6A) was unaffected, however they walked slightly slower (5.4 mm/s) and for shorter periods (1.2 s) with a reduced response to mechanical stimulation (0.8 mm/s). Interestingly, GCaMP imaging of R2 neurons showed no temperature response (0% fluorescence change) and only a small 8% increase in *R78B06* > *GCaMP6f; TrpA1* preparations (Fig. 6B and Supplementary Fig. S3). Of note, *R78B06* > *GCaMP6f; shibire*^*TS*^ flies could not be tested as they did not produce viable offspring. *R78B06* > *trans*-*Tango* identified R2 ring neurons and their layer-specific presynaptic projections to the EB ring neuropil (Fig. 6C, left), with postsynaptic *trans*-Tango labelling detectable in R2 and R4m ring neuron subtypes and neuropil layers (Fig. 6C, middle and right, and D, F), as well as pre-and postsynaptic labelling in specific layers of the FB (Fig. 6E). Together these data demonstrate that R4m but not R2 neurons respond to increasing temperature and mediate thermoresponsive motor behavior in *Drosophila*.

## Discussion

Our results implicate ring neuron circuits of the central complex (CX) as part of the regulatory network underlying thermoresponsive motor behavior in *Drosophila*. The presented findings suggest that specific EB ring neurons receive and integrate external temperature changes above 25 °C. Their subtype-specific functions indicate that segregated ring neuron circuit activities mediate sensory-motor transformation that gates adaptive motor output in *Drosophila*.

### Temperature increase is registered by ring neuron sub-circuit activity

Previous studies showed that temperature changes > 25 °C are detected by peripheral Hot Cell (HC) neurons in the arista which mediate rapid (< 1 min) warmth avoidance in adult flies^11,67^. Anterior Cell (AC) neurons in the adult brain mediate long term warmth avoidance by responding to smaller thermal gradients^7^. In both cases, however, it has remained elusive how these sensory cues of warming-related temperature changes elicit thermoresponsive movements to relocate to more ambient surroundings fitting the thermal preference of *Drosophila*^2,68^. In flies, the mushroom bodies and related dopaminergic projections of the Protocerebral Posterior Lateral (PPL1) cluster neurons have been implicated in thermosensory behaviors^14,15,69,70^, with recent connectomics analyses indicating that second and third order neurons relay thermosensory information from the arista to the so called lateral accessory calyx^71^. These data suggest a neural network for long-term warmth avoidance and conditioning^11,32,69,72^ including the mushroom bodies that have been shown to negatively regulate locomotor activity^73^; it is unknown, however, how these networks mediate sensory-motor transformation for thermoresponsive motor behavior.

Our results demonstrate that ellipsoid body (EB) R2/R4 (*EB1-Gal4*) and R3/R4d (*c232-Gal4*) ring neurons respond to temperature changes of > 25 °C by calcium influx, which we detected by both CaMPARI and GCaMP imaging that was not seen for R1 neurons targeted by *c105-Gal4*. The response of R3/R4d ring neurons was temporarily delayed to that of R2/R4 ring neurons, suggesting that warming above 25 °C causes increased neural activity in these EB ring neuron subtypes, but not in R1. While it has been shown that R1 neurons are characterized by high tonic activity^74^, the lack of a significant calcium response to increased temperature suggests either such sensory input is not relayed onto R1 neurons or their high activity precludes detectable changes with calcium imaging. In contrast, our physiological analysis of EB1-targeted ring neurons indicates that increasing temperature evokes neural activity in a time and temperature-related manner. The further dissection of R2 and R4m neurons revealed that R4m neurons directly respond to warming, which is further supported by the fact that simultaneous expression of *UAS-shibire*^*TS*^ largely abolished the temperature-related GCaMP signal. Furthermore, an increase in GCaMP signal was also observed for R3/R4d ring neurons, suggesting that R3–R4 neurons register temperature changes above 25 °C with enhanced neural activity.

Ring neurons, however, normally do not express warm-sensing thermoreceptors^7,11–13,67,75^. AC neurons that sense warming of > 25 °C have been shown to project to the superior lateral protocerebrum, the antennal lobe and the subesophageal ganglion^7^. Anatomical studies revealed neuronal connections between the superior lateral protocerebrum and the central complex^76^, this raises the possibility that integrated temperature changes are relayed onto ring neurons via such a network. Moreover, recent studies identified connections between AC neurons and PDF-expressing neurons^16^ that are themselves connected to EB ring neurons^77^. We used ex vivo preparations and Ca^2+^-imaging to show that R4m/d and R3 but not R1 neurons specifically respond to warming between 20 and 35 °C. These data together with known connectomics indicate that warm-sensing AC neurons relay temperature changes of > 25 °C via the superior lateral protocerebrum and/or PDF-expressing neurons to R4m/d and R3 neurons, the exact network of which was not revealed by previous studies^78^ nor by the recent hemibrain connectome analysis^79^, and thus remains to be determined.

### Segregated ring neuron activity mediates thermoresponsive motor behavior

**Figure 7:**
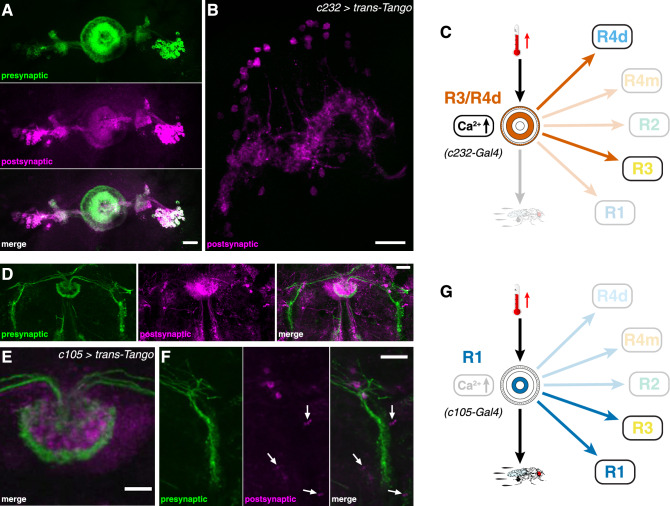
Segregated connectivity and function of R3/R4d and R1 ring neurons in thermoresponsive motor behavior. (**A**,**B**) *trans*-Tango based anterograde transsynaptic tracing of c232-targeted R3/R4d ring neurons indicates reciprocal connections onto themselves and connections to (**B**) columnar wedge neurons but not directly to other rings. (**A**) Detail of presynaptic signal (left top, green), postsynaptic targets (left middle, magenta) and merged image (left bottom) of EB cell bodies and ring neuropil, as well as the bulb. (**C**) R3/R4d ring neurons only connect to themselves; they integrate temperature increase but do not mediate thermoresponsive motor behavior. (**D–F**) c105-targeted *trans*-Tango labelling identifies presynaptic R1 and R3 layers of the EB ring neuropil, as well as the LAL (left, green). Postsynaptic targets (middle, magenta) include R1 and some parts of R3 as seen in the merged image (right), (**E**) detail of the ring structure in a different brain (merged image shown) and (**F**) detail of the LAL connections (arrows). (**G**) R1 ring neurons connect to themselves and R3; they do not integrate temperature increase but mediate thermoresponsive motor behavior. Scale bars, 20 µm.

The observed warming-related calcium influx in R4m/d and R3 neurons suggests they are involved in sensory-motor transformation for thermoresponsive motor behavior. Surprisingly, however, only R4m neurons but not R2 or R4d and R3 neurons showed impaired thermoresponsive motor behavior at 31 °C when they expressed the temperature-sensitive mutant allele of dynamin. *shibire*^*TS*^ has been shown to affect synaptic vesicle endocytosis^52^, thus acting on postsynaptic targets by inhibiting synaptic transmission which in turn can modify behavior^53^. Our observations suggest that R2 as well as R3/R4d ring neurons are either not involved in thermoresponsive behavior or their connectivity network precludes gating of adaptive motor output. *trans*-Tango mediated transsynaptic tracing^63^ identifies R3/R4d ring neurons that connect only onto themselves and to columnar wedge neurons (Fig. 7A–C), and despite the fact that they show warming-related calcium influx, R4d and R3 neurons do not mediate thermoresponsive motor behavior (Fig. 2). Moreover, R2 neurons did not show a significant warming-related calcium influx, nor did expression of *shibire*^*TS*^ at 31 °C impair thermoresponsive motor behavior of *R78B06* > *shibire*^*TS*^ flies (Fig. 6A,B). *trans*-Tango mediated transsynaptic tracing identifies R2 neurons that connect onto themselves and onto R4m neurons. However, in contrast to R2, R4m neurons showed warming-related calcium influx and impaired thermoresponsive motor behavior at 31 °C when simultaneously expressing *shibire*^*TS*^. Together these data indicate that R3/R4d neurons integrate warming-related temperature changes above 25 °C but themselves do not directly mediate thermoresponsive motor behavior.

**Figure 8:**
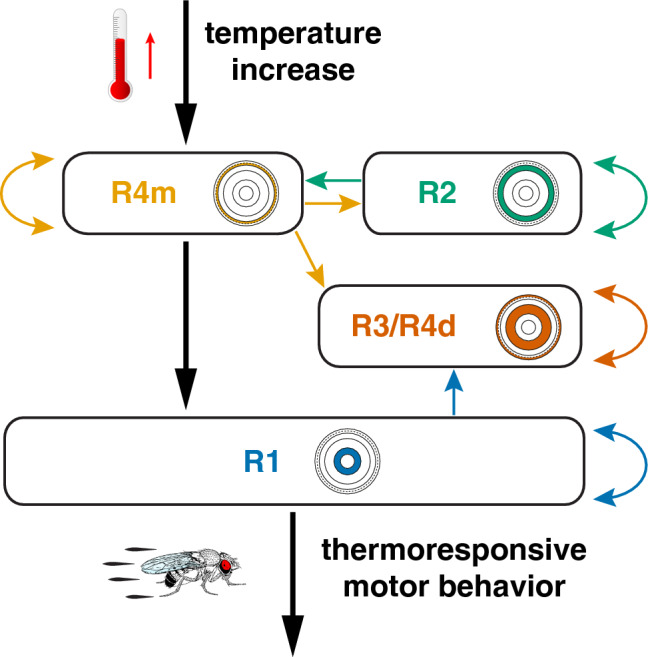
Summary of findings and proposed model. Segregated ring neuron circuit activities mediate sensory-motor transformation whereby R4m neurons integrate and convey warming-related temperature changes above 25 °C to R2–R4 circuits. R1 acts as the bona fide output layer gating thermoresponsive motor behavior in *Drosophila*.

Instead, we observed a strong impairment of warming-related motor output in R1—*c105* > *shi*^*TS*^ flies that was also observed in R2/R4—*EB1* > *shi*^*TS*^ as well as in R4m—*R59B10* > *shi*^*TS*^ flies. *trans*-Tango analysis of these *Gal4* lines revealed that R2/R4 ring neurons as well as R4m neurons connected onto themselves and to all other ring neuron layers, whereas R1 neurons are connected to themselves, R3 and the LAL (Fig. 7D–G), consistent with electron-microscopy (EM) based connectome data^79^. However, our data reveal that R3 neurons did not trigger impairment of warming-related motor output of *c232* > *shi*^*TS*^ flies at 31 °C, which was detected in R1—*c105* > *shi*^*TS*^ flies. Moreover, the fact that R1 neurons did not show alterations in Ca^2+^ influx upon temperature changes above 25 °C, suggests that sensory-motor transformation is distributed among EB ring neurons. The dynamics of the observed calcium influx suggest R4m as the input layer where temperature changes are registered and reverberated with connected R3/R4d layers, as indicated by the temporal delay in Ca^2+^-imaging of *c232* > *GCaMP6f* when compared to R2/R4—*EB1* > *GCamP6f* or R4m—*R59B10* > *GCamP6f* (Supplementary Fig. S3). Thus, *Trans*-Tango related connectivity patterns and *shibire*^*TS*^-triggered behavioral changes suggest that integrated sensory information of warming-related temperature changes is conveyed to R1 as the bona fide output layer gating thermoresponsive motor behavior in *Drosophila* (Fig. 8).

### Ring neuron circuits are anatomical substrates for sensory-motor transformation

Theoretical considerations and CX-related neural network simulations hypothesized that the EB expresses a certain degree of directionality by specialized input and output layers^58,59^. Our findings further suggest specialized layers of the EB ring neuropil that express a concentric directionality from the outer rim as input layer to the R1 center that serves to gate parts of the lateral accessory lobe to control descending pathways. This hypothesis is also supported by EM based connectome data which identify R1 neurons (e.g. ID1291099014) and their postsynaptic connections onto the LAL^79^. Our GCaMP imaging together with electrophysiological recordings revealed a saccade of small peaks starting around 23 °C which by > 30 °C coalesced into a large fluorescent spike (Fig. 3A,D), suggesting that temperature increase triggers ring neuron subtype-specific activity in a threshold-dependent manner. Based on these observations, we speculate that an all-or-nothing response mediates sensory-motor transformation in thermoresponsive motor behavior: Once warming rises above ~ 28 °C, spiking of outer rim layer neurons is relayed onto R1 neurons that gate a motor response. Given the recently identified ring neuron sublayers and shells^79^, it remains to be shown whether segregated ring neuron activity is further distributed into sublayer and/or shell activities.

The observed ring neuron functions in sensory-motor transformation are not restricted to thermoresponsive motor behavior. A number of studies utilizing different behavioral paradigms but targeting subtypes similar to our study showed that ring neuron circuits are involved in a wide range of adaptive behaviors, from visual pattern^33^ and spatial memory formation in a flight simulator^31,44^, to ethanol-related sensitivity^80^ and ethanol-induced locomotion^28^, as well as to startle-induced arousal where flies responded to repeated air puffs^29^. Of note, outer layer ring neurons are known to be inhibitory GABAergic (Refs.^57,60,81^ and Fig. 3) and R1 neurons receive direct GABAergic inhibition^74^. The resulting center-surround inhibition within the ring neuron network is a central feature of ring attractor dynamics underlying spatial navigation in *Drosophila*^82^. Such networks exert winner-takes-all functionality whereby reciprocal inhibition leads to the selection of only one of many signals^83^, which in turn can convey directed output to the next network level^59,84^. The recently identified inhibitory projections between EB and LA^74^ could reverberate the selection process also during persistent stimulus input, such as elevated temperature. As a node of convergence situated several synapses downstream of sensory neurons and several synapses upstream of motor ganglia, the position and connectivity of the EB-LAL interface implies a direct role in the translation of sensory representations into motor representations. Indeed, experiments in locusts^85^ and silk moths^86^ identified the LAL as a premotor command centre projecting to descending neurons that innervate central pattern generators executing motor actions^40^. This conceptual framework is consistent with recent connectome data^79^ and together with our findings indicate that ring neurons of the central complex integrate multiple sensory modalities, including warming-related temperature changes, to gate adaptive motor behavior in *Drosophila*.

## Methods

### *Drosophila* lines

Flies were maintained on standard cornmeal medium at 25 °C in a 12 h:12 h light/dark cycle. The following strains were obtained from the Bloomington stock center: ellipsoid body (EB) ring specific *Gal4* lines R2/R4—*EB1-Gal4* (BL44409), R2—*R78B06-Gal4* (BL48343), R4m—*R59B10-Gal4* (BL70753), R3/R4d—*c232-Gal4* (BL30828), R1—*c105-Gal4* (BL30822); For functional imaging, *UAS-GCaMP6f* (BL52869) or *UAS-CaMPARI* (BL58761), for synaptic tracing, *UAS-trans-Tango* (gift from Dr James Jepson, UCL) and for thermogenetic activation and inhibition, *UAS-TrpA1* (BL27593) and *UAS-shibire*^*TS*^ (gift from Dr James Jepson, UCL) were combined with the R neuron-specific *Gal4* lines; For behavioral experiments, controls were generated by crossing driver and responder lines to *w1118*; For electrophysiological recordings, *UAS-mCD8::GFP* (BL5137) was crossed to *EB1-Gal4*.

### Behavioral analysis

Three to five day old female flies were individually placed in an open-field arena and their behavior recorded with a Logitech c920 camera at 10 frames per second. The position of the flies was extracted on every other frame (0.2 s) with the DART software^47^. Experiments were performed on custom-made platforms made of an acetal copolymer (POM-C), Tecaform AH. A platform comprised 36 open-field arenas, each arena 3.5 cm in diameter and 1.5 mm high, all covered with a transparent acrylic sheet with 0.1 mm breathing holes. The platform was placed on a white light plate that provided uniform cold light illumination within a temperature-controlled incubator (Stuart Scientific). 36 flies, 12 of each genotype, were simultaneously recorded for 1 h. After 30 min, a mechanical stimulus, composed of 5 vibrations at 3 V for 200 ms separated by 800 ms, were delivered through shaft-less motors (Precision Microdrives) controlled by the DART software. The behavioral experiments were carried out at either 25 °C or 31 °C. Flies were separated at least 24 h prior to the experiment and on the day of the experiment, after a brief cold anaesthesia, immediately transferred individually to their arena. Flies were left to acclimatise and accommodate for 60 min before the experiment. Video-assisted motion tracking and analysis was carried out using the DART system as described previously^45,47^.

### *trans*-Tango mediated transsynaptic tracing

Connectivity of the various rings was investigated with the *trans*-Tango transsynaptic tracing system^63^. Adult female *Drosophila* brains were immuno-stained as described previously^45^. Brains were fixed in PBS with 4% paraformaldehyde at RT for 1 h and then blocked in PBT plus 2% NGS for 20 min. Pre-synaptic neurons were labelled with GFP (rabbit polyclonal anti-GFP, 1:1000; Thermo Fisher Scientific A-11122; RRID: AB_221569) and post-synaptic neurons with mtdTomato (mouse monoclonal anti-HA, 1:250; Covance MMS-101P; clone# HA.11; RRID: AB_2314672). Incubation lasted overnight at 4 °C. After three 10 min PBT washes, brains were incubated for 2 h with secondary antibodies at RT. The secondary antibodies were donkey anti-rabbit conjugated to Alexa Fluor 488 (1:150; Thermo Fisher Scientific A-21206; RRID: AB_2535792) and donkey anti-mouse conjugated to Alexa Fluor 555 (1:150; Thermo Fisher Scientific A-31570; RRID: AB_2536180). Tissues were mounted in Vectashield (Vector Laboratories). Images were acquired with a Nikon A1R confocal microscope equipped with 40 × 1.3NA Plan Fluor oil immersion objective and processed using the Fiji software.

### Functional imaging and electrophysiology

All experiments were performed on two to five day old flies of either sex and brains dissected as described earlier^87^ in extracellular saline solution containing (in mM): 101 NaCl, 1 CaCl_2_, 4 MgCl_2_, 3 KCl, 5 glucose, 1.25 NaH_2_PO_4_, 20.7 NaHCO_3_, pH adjusted to 7.2. Brains were placed in a temperature-controlled recording chamber using a Peltier heating system (ALA Scientific Instruments, NY, USA) and TC-10 controller (npi, Tamm, Germany) on an upright Zeiss microscope (Examiner.Z1, Carl Zeiss Microscopy GmbH, Jena, Germany) with a Colibri light source (365 nm, 470 nm and 555 nm LED).

#### CaMPARI

Response of EB ring neurons to elevated temperature in intact animals was measured using the fluorescent ratiometric calcium sensor CaMPARI^48^. Single flies were kept in either 25 °C or 31 °C for 30 min prior to the experiment. Flies were then quickly beheaded, their brains dissected in prewarmed Ca^2+^ free external solution and placed in a preheated recording chamber. For photoconversion, 20 pulses of 5 s duration and with a 10 s interval were delivered to the whole brain with a 365 nm LED (3.96 mW/cm^2^) using a 20 × lens. Images of the green (470 nm, 1.41 mW/cm^2^) and red channel (555 nm, 2.55 mW/cm^2^) were acquired immediately using an optiMOS camera (QImaging, Surrey, BC, Canada) with exposure times adjusted depending on the staining intensity ranging from 1 to 3 s and kept the same for both channels. A stack of 2.5 µm thick virtual slices encompassing the whole central complex including ring structure and cell bodies was used to produce a maximum intensity projection and the ratio between the two channels for a region of interest drawn around the ring was used for analysis (Zeiss ZEN and Fiji 2.1.0^88^ software).

#### GCaMP

To test the temporal sequence of the Ca^2+^ response to elevated temperature in the central complex we utilised GCaMP6f^54^. Whole brains were gradually heated from 20 to 35 °C and cooled back down to 20 °C and the calcium fluorescence signal obtained using a CCD camera (Zeiss Axiocam), a 470 nm LED light source (3.04 mW/cm^2^) and a 20 × water immersion lens. Images were acquired at 4 fps with 15–50 ms exposure, recorded with ZEN (Zeiss) and plotted with Microsoft Excel. Baseline fluorescence (F_0_) was taken as the mean fluorescence of the first 5 images before any temperature change. The change in fluorescence relative to baseline (ΔF/F_0_) was recorded and the peak change used as a metric of the transient Ca^2+^ increase.

#### Whole-cell recordings

Current clamp recordings were performed as described^87^ using glass electrodes with 8–15 MΩ resistance filled with intracellular solution (in mM: 102 K-gluconate, 17 NaCl, 0.94 EGTA, 8.5 HEPES, 0.085 CaCl_2_, 1.7 MgCl_2_, pH 7.2) and an Axon MultiClamp 700B amplifier, digitised with an Axon DigiData 1440A (sampling rate: 20 kHz; filter: Bessel 10 kHz) and recorded using pClamp 10 (Molecular Devices, CA, USA). After cleaning the brain, a small incision was made over the position of the EB1 neurons in order to give easier access for the recording electrode. Brains were placed ventral side up in the temperature-controlled recording chamber, secured using a custom-made anchor and continuously perfused with aerated (95% O_2_, 5% CO_2_) saline solution. Picrotoxin (Sigma, 250 µM in extracellular solution) was bath applied through the perfusion system. The liquid junction potential was calculated as 13 mV and subtracted from all membrane voltages.

### Statistical analysis

Each dataset was tested for normality using the D’Agostino & Pearson test or the Shapiro–Wilk test for small N, with alpha = 0.05 and then either parametric or non-parametric tests used as appropriate. For behavioral experiments, a one-way ANOVA with either Sidak’s or Dunnett’s post hoc test, a t-test, a Mann–Whitney test or Kruskal–Wallis with Dunn’s post hoc test were used as indicated in figure legends. For each test group, two controls (*Gal4* and *UAS* parental lines) were used and significance only reported if different to both. Data are presented either as Solid lines (mean) with shaded area (standard error of the mean, SEM), box (median and interquartile range, IQR) and whisker (Tukey) plots or bar charts (mean, SEM). For imaging experiments, a one-way ANOVA with Sidak’s post hoc test for CaMPARI; a one-sample t-test or Wilcoxon Signed Rank test for the GCaMP response to heat; a one-way ANOVA with Dunnett’s post hoc test, a Kruskal–Wallis with Dunn’s post hoc test or a Mann–Whitney test for GCaMP comparison with *TrpA1* and *shibire*^*TS*^ were used. Data are presented as mean and standard deviation (SD) and individual data points displayed. All statistical tests were performed using GraphPad Prism and all test results are presented in Supplementary Table S1. Figures were arranged in Adobe Illustrator.

## Supplementary Information

Supplementary Information.

Supplementary Video 1.
