# Supplementary material for: Thermoresponsive motor behavior is mediated by ring neuron circuits in the central complex of Drosophila
Source: Sci Rep. 2021 Jan 8;11:155. doi: 10.1038/s41598-020-80103-9 (PMC7794218; doi:10.1038/s41598-020-80103-9)
Supplement: Supplementary file 1 — Supplementary Information. [file 41598_2020_80103_MOESM1_ESM.pdf]

Thermoresponsive motor behavior is mediated by ring neuron circuits in the central complex of *Drosophila*

Edgar Buhl, Benjamin Kottler, James J.L. Hodge and Frank Hirth

Supplementary Material

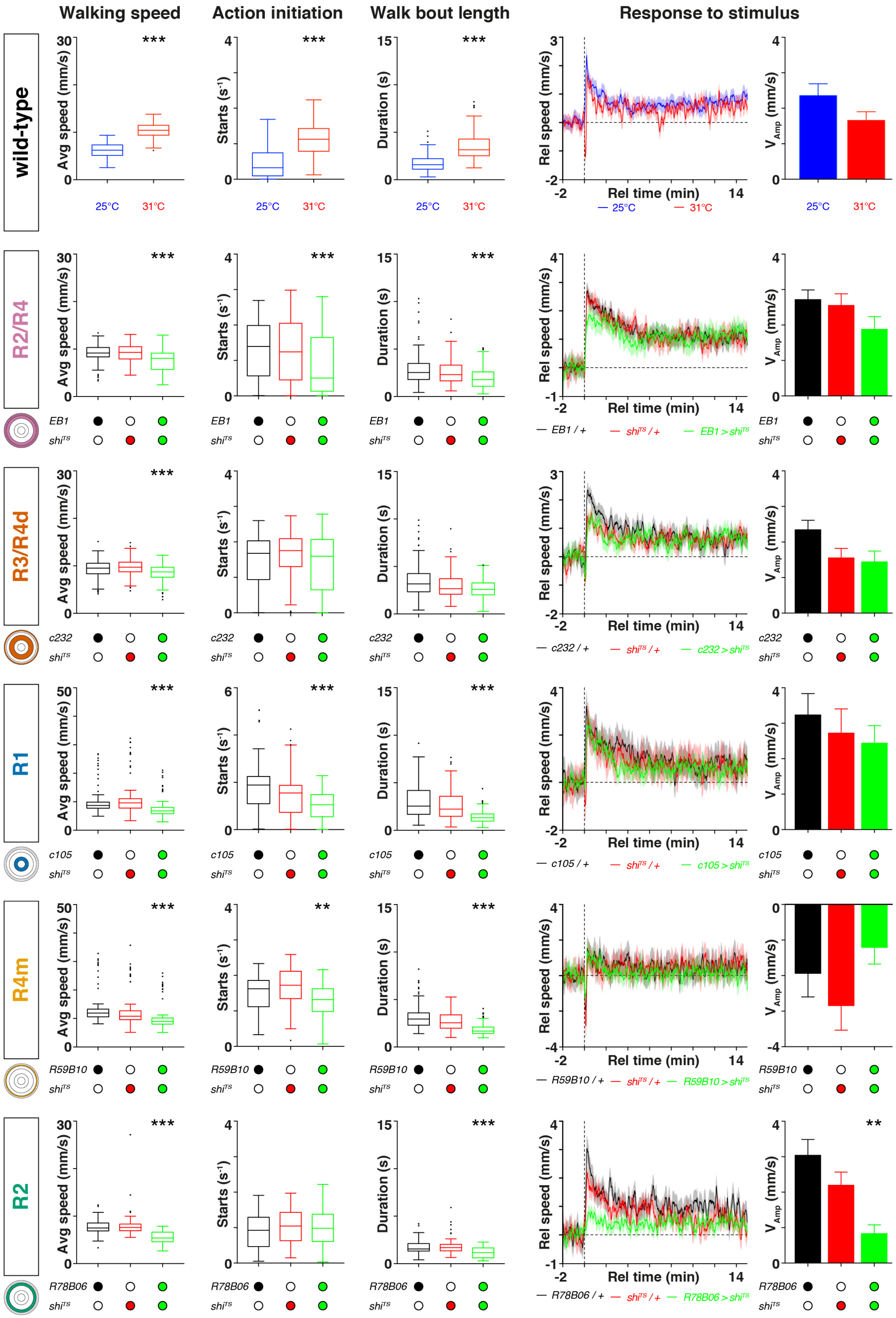

Walking speed
Action initiation
Walk bout length
Response to stimulus
wild-type
R2/R4
R3/R4d
R1
R4m
R2
Avg speed (mm/s)
Starts (s⁻¹)
Duration (s)
Rel speed (mm/s)
V Amp (mm/s)
Rel time (min)
25°C
31°C
***
**
EB1
c232
c105
R59B10
R78B06
shiTS
EB1 / +
shiTS / +
EB1 > shiTS
c232 / +
c232 > shiTS
c105 / +
c105 > shiTS
R59B10 / +
R59B10 > shiTS
R78B06 / +
R78B06 > shiTS

Figure S1: Walking, initiation and stimulus response behavioral parameters for temperature-induced locomotion. *Related to Figures 1, 2, 5 and 6.*

Average active walking speed, number of action initiations and average activity bout length as well as walking speed change in response to stimulation (V_{Amp}) for controls and all tested genotypes with *shibire*^{TS}. Box and whiskers, median, IQR, Tukey; bars and whiskers, mean, SEM. * p<0.05, ** p<0.01, *** p>0.001, ANOVA with Sidak's *post hoc* test for all stim responses; ANOVA with Dunnett's *post hoc* test for R4m *shibire*^{TS} initiations; Mann-Whitney test for wild-type except t-test for speed and stim response; Kruskal-Wallis with Dunn's test for all others.

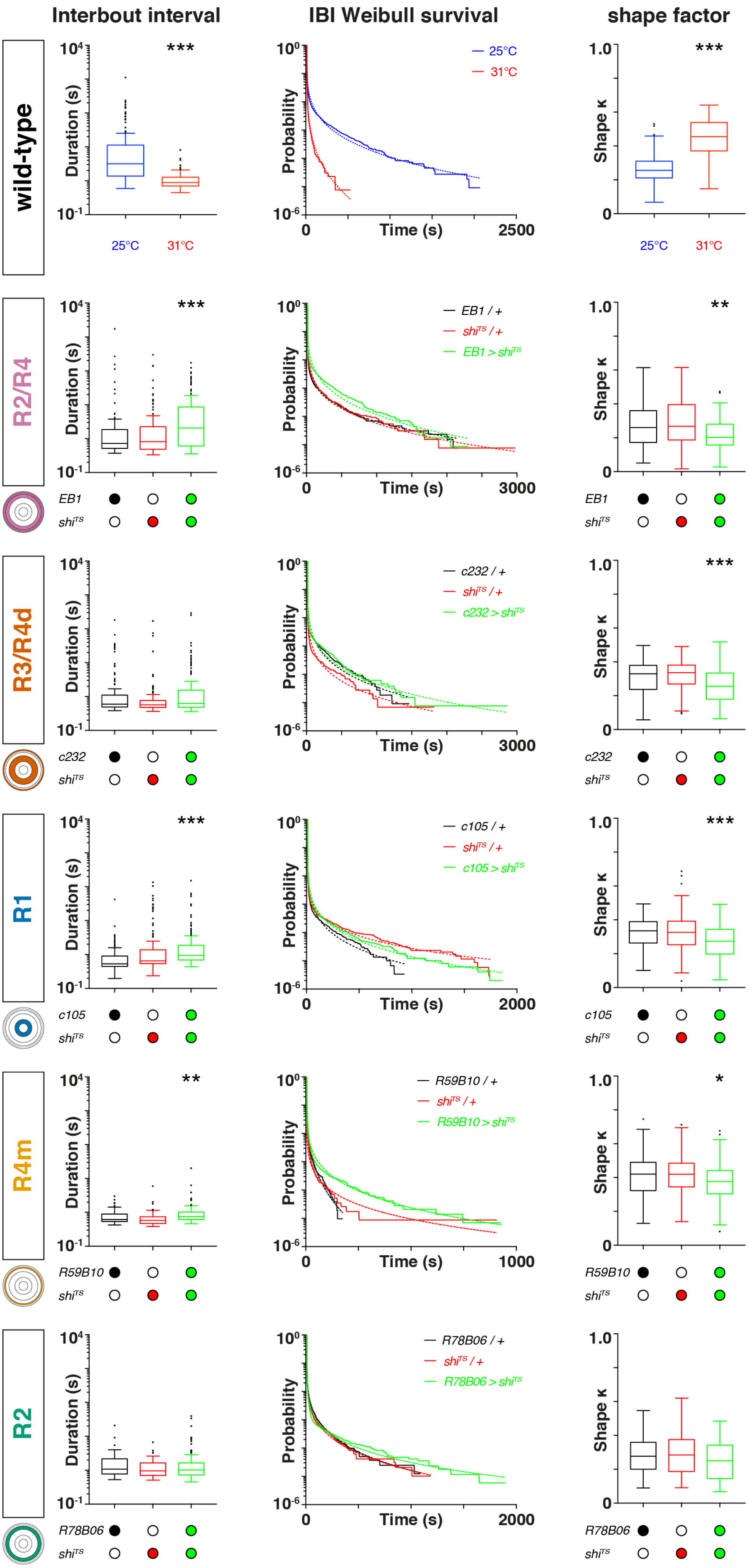

Interbout interval
IBI Weibull survival
shape factor
wild-type
R2/R4
R3/R4d
R1
R4m
R2
Duration (s)
Probability
Shape κ
Time (s)
25°C
31°C
EB1 / +
shiTS / +
EB1 > shiTS
c232 / +
c232 > shiTS
c105 / +
c105 > shiTS
R59B10 / +
R59B10 > shiTS
R78B06 / +
R78B06 > shiTS
EB1
c232
c105
R59B10
R78B06
shiTS

Figure S2: Interbout-interval analysis for temperature-induced locomotion. *Related to Figures 1, 2, 5 and 6.*

Inter-bout interval (IBI), cumulative Weibull curves (solid line, raw data; dashed line, model) and shape factor κ for controls and all tested genotypes with *shibire*^{TS}. Note, axes for all IBIs are in logarithmic scale (log_{10}). Box and whiskers, median, IQR, Tukey. ** p<0.01, *** p>0.001, ANOVA with Dunnett's *post hoc* test for R4m *shibire*^{TS} shape factor; Mann-Whitney test for wild-type; Kruskal-Wallis with Dunn's test for all others.

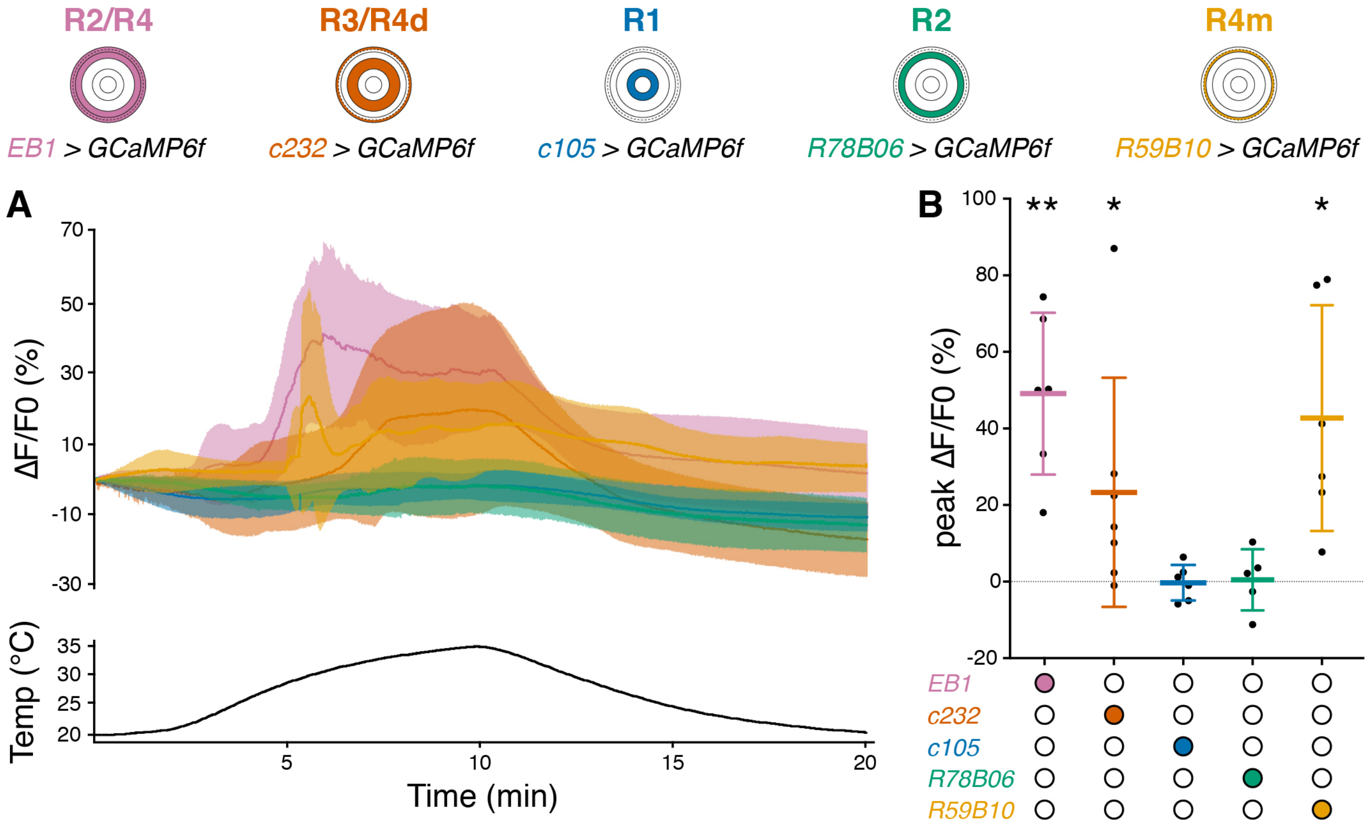

Figure S3: Increasing temperature above 25°C elicits ring neuron subtype-specific Ca^{2+} response. *Related to Figures 2, 3, 5, 6 and video SV1.*

(**A**) Average GCaMP imaging of R2/R4 (*EB1-Gal4*, reddish purple), R3/R4d (*c232-Gal4*, vermilion), R1 (*c105-Gal4*, blue), R2 (*R78B06-Gal4*, bluish green) and R4m (*R59B10-Gal4*, orange) ring neurons in response to temperature ramps from 20°C to 35°C as measured in the ring neuropil. Note that the onset of the Ca^{2+} response of R3/R4d is delayed (at higher temperatures) when compared to R2/R4 or R4m.

(**B**) Peak GCaMP signals demonstrating a Ca^{2+} response for R2/R4, R3/R4d and R4m but not for R1 and R2. Note, data are replotted at different scale from the controls in Figures 2D-F, 5B and 6B (black graphs). Mean ± SD, n≥5. * $p<0.05$, ** $p>0.01$, one-sample t-test or Wilcoxon Signed Rank test compared to no response, i.e. 0.

Video SV1: Temperature induced Ca^{2+} response EB ring neurons. *Related to Figure 3.* Video (4 fps, sped up 6 times) of an exemplary GCaMP response of *EB1-Gal4* specific R2/R4 neurons to a temperature ramp from 24°C to 38°C. Note, video is focussed on the cell body layer (individual neurons on left and right side of the video) and the ring neuropil is in the middle out of focus.

Table S1: Statistical test results. *Related to Figures 1, 2, 5, 6, S1, S2 and S3.*

Gal4 driver	Comparison	p-value	Normality	Statistical test
**Activity**				
wild-type	25°C vs 31°C	<0.0001	no	Mann-Whitney
EB1>shibire^{TS}	*EB1 / +*	<0.0001	no	Kruskal-Wallis with Dunn's
	shibire^{TS} / +	<0.0001		
c232>shibire^{TS}	*c232 / +*	0.0072	no	Kruskal-Wallis with Dunn's
	shibire^{TS} / +	0.1676		
c105>shibire^{TS}	c105 / +	<0.0001	no	Kruskal-Wallis with Dunn's
	shibire^{TS} / +	<0.0001		
R59B10>shibire^{TS}	*R59B10 / +*	<0.0001	no	Kruskal-Wallis with Dunn's
	shibire^{TS} / +	<0.0001		
R78B06>shibire^{TS}	*R78B06 / +*	0.1126	no	Kruskal-Wallis with Dunn's
	shibire^{TS} / +	0.0011		
**Walking speed**				
wild-type	25°C vs 31°C	<0.0001	yes	t-test
EB1>shibire^{TS}	*EB1 / +*	<0.0001	no	Kruskal-Wallis with Dunn's
	shibire^{TS} / +	<0.0001		
c232>shibire^{TS}	*c232 / +*	0.0005	no	Kruskal-Wallis with Dunn's
	shibire^{TS} / +	<0.0001		
c105>shibire^{TS}	c105 / +	<0.0001	no	Kruskal-Wallis with Dunn's
	shibire^{TS} / +	<0.0001		
R59B10>shibire^{TS}	*R59B10 / +*	<0.0001	no	Kruskal-Wallis with Dunn's
	shibire^{TS} / +	<0.0001		
R78B06>shibire^{TS}	*R78B06 / +*	<0.0001	no	Kruskal-Wallis with Dunn's
	shibire^{TS} / +	<0.0001		
**Action initiation**				
wild-type	25°C vs 31°C	<0.0001	no	Mann-Whitney
EB1>shibire^{TS}	*EB1 / +*	<0.0001	no	Kruskal-Wallis with Dunn's
	shibire^{TS} / +	0.0003		
c232>shibire^{TS}	*c232 / +*	0.9240	no	Kruskal-Wallis with Dunn's
	shibire^{TS} / +	0.0455		
c105>shibire^{TS}	c105 / +	<0.0001	no	Kruskal-Wallis with Dunn's
	shibire^{TS} / +	<0.0001		
R59B10>shibire^{TS}	*R59B10 / +*	0.0027	yes	ANOVA with Dunnett's
	shibire^{TS} / +	0.0001		
R78B06>shibire^{TS}	*R78B06 / +*	0.6685	no	Kruskal-Wallis with Dunn's
	shibire^{TS} / +	>0.9999		
**Walk bout length**				
wild-type	25°C vs 31°C	<0.0001	no	Mann-Whitney
EB1>shibire^{TS}	*EB1 / +*	<0.0001	no	Kruskal-Wallis with Dunn's
	shibire^{TS} / +	<0.0001		
c232>shibire^{TS}	*c232 / +*	0.0003	no	Kruskal-Wallis with Dunn's
	shibire^{TS} / +	0.4430		
c105>shibire^{TS}	c105 / +	<0.0001	no	Kruskal-Wallis with Dunn's

	shibire^{TS} */ +*	<0.0001		
R59B10>shibire^{TS}	*R59B10 / +*	<0.0001	no	Kruskal-Wallis with Dunn's
	shibire^{TS} */ +*	<0.0001		
R78B06>shibire^{TS}	*R78B06 / +*	<0.0001	no	Kruskal-Wallis with Dunn's
	shibire^{TS} */ +*	<0.0001		

Response to stimulus

wild-type	25°C vs 31°C	0.0942	yes	t-test
EB1>shibire^{TS}	*EB1 / +*	0.1198	yes	ANOVA with Dunnett's
	shibire^{TS} */ +*	0.2415		
c232>shibire^{TS}	*c232 / +*	0.0465	yes	ANOVA with Dunnett's
	shibire^{TS} */ +*	0.9434		
c105>shibire^{TS}	c105 / +	0.5433	yes	ANOVA with Dunnett's
	shibire^{TS} */ +*	0.9164		
R59B10>shibire^{TS}	*R59B10 / +*	0.6058	yes	ANOVA with Dunnett's
	shibire^{TS} */ +*	0.1078		
R78B06>shibire^{TS}	*R78B06 / +*	<0.0001	yes	ANOVA with Dunnett's
	shibire^{TS} */ +*	0.0080		

Interbout interval

wild-type	25°C vs 31°C	<0.0001	no	Mann-Whitney
EB1>shibire^{TS}	*EB1 / +*	0.0002	no	Kruskal-Wallis with Dunn's
	shibire^{TS} */ +*	0.0003		
c232>shibire^{TS}	*c232 / +*	0.9190	no	Kruskal-Wallis with Dunn's
	shibire^{TS} */ +*	0.0462		
c105>shibire^{TS}	c105 / +	<0.0001	no	Kruskal-Wallis with Dunn's
	shibire^{TS} */ +*	<0.0001		
R59B10>shibire^{TS}	*R59B10 / +*	0.0022	no	Kruskal-Wallis with Dunn's
	shibire^{TS} */ +*	<0.0001		
R78B06>shibire^{TS}	*R78B06 / +*	0.6840	no	Kruskal-Wallis with Dunn's
	shibire^{TS} */ +*	>0.9999		

IBI Weibull shape factor

wild-type	25°C vs 31°C	<0.0001	no	Mann-Whitney
EB1>shibire^{TS}	*EB1 / +*	0.0018	no	Kruskal-Wallis with Dunn's
	shibire^{TS} */ +*	<0.0001		
c232>shibire^{TS}	*c232 / +*	0.0003	no	Kruskal-Wallis with Dunn's
	shibire^{TS} */ +*	<0.0001		
c105>shibire^{TS}	c105 / +	<0.0001	no	Kruskal-Wallis with Dunn's
	shibire^{TS} */ +*	0.0002		
R59B10>shibire^{TS}	*R59B10 / +*	0.0254	yes	ANOVA with Dunnett's
	shibire^{TS} */ +*	0.0095		
R78B06>shibire^{TS}	*R78B06 / +*	0.1087	no	Kruskal-Wallis with Dunn's
	shibire^{TS} */ +*	0.1757		

CaMPARI

EB1>CaMPARI	25°C vs 31°C	<0.0001	yes	ANOVA with Sidak's
c232>CaMPARI	25°C vs 31°C	0.0034	yes	ANOVA with Sidak's
c105>CaMPARI	25°C vs 31°C	0.9482	yes	ANOVA with Sidak's

**GCaMP**				
EB1>GCaMP6f	peak vs no response	0.0023	yes	t-test
c232>GCaMP6f	peak vs no response	0.0312	no	Wilcoxon Signed Rank
c105>GCaMP6f	peak vs no response	0.8828	yes	t-test
R59B10>GCaMP6f	peak vs no response	0.0165	yes	t-test
R78B06>GCaMP6f	peak vs no response	0.9060	yes	t-test
EB1>GCaMP6f	*EB1>GCaMP6f; TrpA1*	0.0001	yes	ANOVA with Dunnett's
	EB1>GCaMP6f; shibire^{TS}	0.0001	yes	ANOVA with Dunnett's
c232>GCaMP6f	*c232>GCaMP6f; TrpA1*	0.0075	no	Kruskal-Wallis with Dunn's
	c232>GCaMP6f; shibire^{TS}	>0.9999	no	Kruskal-Wallis with Dunn's
c105>GCaMP6f	*c105>GCaMP6f; TrpA1*	0.3140	yes	ANOVA with Dunnett's
	c105>GCaMP6f; shibire^{TS}	0.2175	yes	ANOVA with Dunnett's
R59B10>GCaMP6f	*R59B10>GCaMP6f; TrpA1*	0.9800	yes	ANOVA with Dunnett's
	R59B10>GCaMP6f; shibire^{TS}	0.1448	yes	ANOVA with Dunnett's
R78B06>GCaMP6f	*R78B06>GCaMP6f; TrpA1*	0.2468	no	Mann-Whitney
